# Curbing the Epidemic of Community Firearm Violence after the *Bruen* Decision

**DOI:** 10.1017/jme.2023.42

**Published:** 2023

**Authors:** Jonathan Jay, Kalice Allen

**Affiliations:** 1. BOSTON UNIVERSITY SCHOOL OF PUBLIC HEALTH, BOSTON, MASSACHUSETTS, USA

**Keywords:** Firearms, Violence Prevention, Structural Racism, Racial Segregation, Gun Control

## Abstract

The Supreme Court’s decision in *New York State Rifle & Pistol Association Inc. v. Bruen* undermines the ability of cities and states to regulate firearms safety. Nonetheless, we remain hopeful that firearm violence can decline even after the *Bruen* decision. Several promising public health approaches have gained broader adoption in recent years. This essay examines the key drivers of community firearm violence and reviews promising strategies to reverse those conditions, including community violence intervention (CVI) programs and place-based and structural interventions.

With its decision in *New York State Rifle & Pistol Association Inc. v. Bruen*, the Supreme Court constrained local jurisdictions’ ability to regulate firearm carriage in public settings.^1^ According to *Bruen,* the Second Amendment establishes a right for “law-abiding citizens” to carry firearms outside the home for self-defense. Most immediately, this ruling endangered regulations in six states that enabled officials to deny concealed carry permits to applicants who lacked a special need for the permit, typically related to self-defense. The Court’s decision thereby removed an important regulatory lever that some cities and states had used to limit firearm carriage.

*Bruen* clearly sends jurisprudence in the wrong direction with respect to firearms safety. Research indicates that the expansion of concealed carry is associated with increased firearm violence.^2^ Moreover, the Court held in *Bruen* that only firearm regulations consistent with U.S. historical traditions are compatible with the Second Amendment.^3^ This reasoning could be used to invalidate a range of other modern firearm regulations, enabling easier access to more-lethal weapons.^4^ The timing of this decision is unusually poor: firearm violence rates, already rising before COVID-19, have taken a historic spike since the start of the pandemic.^5^

Nonetheless, we remain hopeful that firearm violence can decline even after the *Bruen* decision. Our national dialogue, centered on the politics of firearm regulations, too often overlooks other approaches to addressing firearm violence. Several promising public health approaches have gained broader adoption in recent years. Compared to firearm regulations, these approaches are better tailored to the social dynamics that drive most firearm violence. Thus, despite the setback that *Bruen* represents, important progress is occurring, especially in the most-impacted communities. This essay examines the key drivers of community firearm violence and reviews promising strategies to reverse those conditions that are not hindered by *Bruen* and the current trajectory of this Supreme Court’s Second Amendment jurisprudence.

Differences in firearm violence are considerably steeper across neighborhood social conditions than across state regulatory conditions — a reminder that permitting requirements are not the most critical determinant of community firearm violence. Instead, the vast disparities across levels of racialized economic segregation demand a focus on the conditions that concentrate firearm violence in the most-deprived neighborhoods.

## Drivers of Community Firearm Violence

Community firearm violence is truly a public health crisis in the United States. For children and teens, firearms have recently become the leading cause of death,^6^ owing mostly to homicides.^7^ Across all ages, a minority of firearm homicides results from intimate partner violence, family violence, school shootings, and mass shootings.^8^ A substantial majority occurs outside the home, between individuals who are not related or romantically involved. This form of interpersonal violence is referred to as “community violence.” Most often, community violence results from routine disputes between individuals.^9^ Thus, a disproportionate burden of the firearm violence epidemic arises from the sort of scenario that concealed carry restrictions (those overturned by *Bruen*) could conceivably help prevent: a public encounter that becomes lethal because one or more parties have ready access to a gun during a moment of interpersonal conflict.

However, limitations on concealed carry are neither necessary nor sufficient to achieve low rates of firearm violence. For instance, one well-designed study examined changes in firearm assault rates before and after changes in concealed carry rules. The authors estimated that firearm assaults increased by approximately 10% over ten years in states switching from the more restrictive may-issue to the less restrictive shall-issue licensing regime.^10^ This finding suggests that loosening concealed carry rules may increase firearm access and thereby contribute to violence, but these effects are modest. Looking more broadly at firearm regulations, Crifasi and colleagues found that in urban counties, where most firearm homicides occur, a handful of laws were associated with higher or lower firearm homicide rates. The estimated effects ranged from a 14% reduction in firearm homicides (from permit-to-purchase requirements) to a 16% increase (from background checks without permitting requirements).

These effect sizes are not inconsequential, but they are eclipsed by other factors. For instance, two studies have analyzed firearm violence as a function of racialized economic segregation, using an indicator called the index of concentration at the extremes (ICE) for race-poverty.^11^ The ICE is a simple, neighborhood-level measure of exposure to structural racism that captures the two most essential, interrelated dimensions of social stratification: by race and class. At the low end of this measure (values closer to -1) are deprived neighborhoods where Black-headed households, with incomes below the poverty line, outnumber White-headed households with incomes over $100,000/year. At the other end (values near 1) are privileged neighborhoods where those proportions are reversed.

Both studies found that racialized economic segregation is an exceptionally strong predictor of firearm assaults. Krieger and colleagues found that living in a neighborhood in the most-deprived ICE quintile was associated with 3.96 times greater risk of a firearm assault injury, compared to living in the most-privileged ICE quintile.^12^ (In other words, a 396% increase in risk.) Their data came from Massachusetts, a may-issue state. Jay and colleagues used data from six U.S. cities, located both in may-issue (Maryland, Delaware, New York) and shall-issue (Pennsylvania, District of Columbia, Virginia) states.^13^ They found that neighborhoods in the most-deprived ICE quintile experienced an average of 36.1 shootings over a six-year period, while neighborhoods in the most-privileged quintile experienced just 2.9. A sizeable ICE-firearm violence association persisted even after controlling for other neighborhood-level factors, such as liquor stores and unemployment rates.

Differences in firearm violence are considerably steeper across neighborhood social conditions than across state regulatory conditions — a reminder that permitting requirements are not the most critical determinant of community firearm violence. Instead, the vast disparities across levels of racialized economic segregation demand a focus on the conditions that concentrate firearm violence in the most-deprived neighborhoods.

## Drivers of Chronic Violence

The drivers of firearm violence are complex and interrelated. Most individuals who enact firearm violence have previously experienced repeated exposure to violence as a victim or witness.^14^ Affiliating with peers makes some young people feel safer, but these social links can create obligations to retaliate when a friend is assaulted.^15^ Community members rally to keep young people safe, but this task is especially challenging in places deprived of resources like high-quality parks, schools, and jobs. An abundance of hazards like liquor stores, vacant lots, or unwalkable streets may further reinforce violence patterns because they hinder positive social interactions.^16^ Each of these processes contributes to vicious cycles, or feedback loops, in which every occurrence of violence makes future violence more likely.

Accordingly, firearm carriage is as much a symptom as a cause of firearm violence. In a sample of youth living in deprived neighborhoods, Sokol and colleagues found that youth’s perceptions of community violence were the single strongest predictor of whether they carried firearms.^17^ In other words, youth carried firearms because they felt vulnerable to serious victimization. This dynamic represents another feedback loop by which violence begets more violence. However, firearm carriage was also entangled with other drivers of violence: youth were more likely to carry firearms when they reported greater victimization, endorsed retaliatory attitudes and had negative peer influences.^18^

These factors are all linked to ICE because segregation enables public and private entities to disinvest from communities of color, especially Black communities.^19^ While firearm violence does occur in neighborhoods with racial and economic privilege, it occurs chronically in neighborhoods characterized by resource gaps. Simply increasing firearm carriage, as shall-issue regulations appear to,^20^ may not be enough to tip a community into chronic firearm violence. Conversely, in the settings most impacted by community firearm violence, targeting firearms alone is not enough to unravel violence patterns. Instead, the two most promising approaches — community violence intervention and place-based or structural interventions — address the disinvestment, from both people and places, that allows firearm violence cycles to persist.

## Community Violence Intervention

Community violence intervention (CVI) describes a category of interventions that aim to halt cycles of violence and promote healing through community-based service provision. CVI models vary, but each relies on “credible messengers”: trained, frontline workers who can overcome clients’ distrust of institutions because they share common experiences, such as growing up in the same neighborhood or navigating the aftermath of violence involvement. Some CVI models, such as Advance Peace, center on mentorship.^21^ Others, including hospital-based violence intervention programs (HVIP), aim to meet clients’ basic needs through trauma-informed, wraparound services.^22^ Another common approach, known as the Cure Violence or “violence interrupter” model, focuses on mediating disputes in the short term and changing norms in the long term.^23^ In practice, CVI programs may combine elements of each of these models, plus counseling or other components.^24^

The evidence for these strategies is encouraging, though still emerging. Implementation of Advance Peace was associated with a 55% reduction in firearm homicides over several years in Richmond, California, but also a concomitant increase in non-firearm violence.^25^ Studies of HVIP often rely on small samples, but program effects point in the right direction for reducing future violent injuries.^26^ Violence interrupter programs have shown benefits^27^ homicide and nonfatal shootings are the leading causes of mortality and morbidity. Urban youth’s attitudes and perceptions about the use of gun violence to resolve conflict present a major barrier to efforts to reduce gun homicides and nonfatal shootings. The current investigation extends the existing literature on attitudes toward guns and shootings among high-risk youth ages 18 to 24 by measuring perceived norms and viewpoints regarding gun violence in two analogous Baltimore City neighborhoods pre-implementation and 1-year post-implementation of the Safe Streets intervention (adapted from the CeaseFire/Cure Violence intervention and some prevention effects^28^ but have proven difficult to evaluate for technical and operational reasons.^29^ The interrupter model retains strong appeal, partly because locally-credible street workers offer the most visible alternative to police in communities where trust in police is low. Interruption is almost certainly more cost-effective than policing^30^ and does not involve the law enforcement contacts that generate over 1,000 killings by police officers each year,^31^ along with mental and physical harms observed among people exposed to “proactive policing” tactics.^32^

## Place-Based and Structural Interventions

Reducing disparities requires not just individual-level interventions, but systematic changes to the contexts where people live, work, study, and play.^33^ Racially and economically marginalized residents are disproportionately exposed to hazardous conditions in the physical and social environment, largely due to disinvestment. Historical redlining is one well-studied example: not only did redlining reinforce *de facto* segregation, but it is also associated with present-day markers of disinvestment, such as less green space^34^ and a higher density of liquor stores.^35^ Each of these factors is independently associated with firearm violence^36^ and may contribute to the relation between redlining and firearm violence that recent studies have found.^37^ Policymakers can begin to reverse these patterns through present-day investments in neighborhood conditions.

Improving the physical environment is one crucial intervention point. Restoring a neighborhood’s physical conditions by creating green spaces,^38^ abandoned property remediations,^39^ or eliminating unmaintained vacant properties^40^ has been found to reduce community gun violence in strong experimental and quasi-experimental studies. A recent randomized controlled trial by South and colleagues found that simple improvements to abandoned houses — installing proper doors and windows and cleaning up the property — produced a 13% reduction in gun assaults and a 7% reduction in shootings in low-income, predominantly Black neighborhoods.^41^ These reductions are striking because they resulted from a low-cost intervention that can be readily combined with other neighborhood-level efforts. A built environment-focused approach may even align with a climate justice agenda: hotter temperatures are associated with more firearm violence,^42^ while investments such as tree canopy, which mitigates heat, are associated with less firearm violence.^43^

Social resources are another area where investments are directly tied to violence. One study found that over time, low-income neighborhoods with more activity from community nonprofits experienced less violence.^44^ A particularly important gap in low-ICE neighborhoods is low access to quality jobs. Researchers have found that large-scale jobs programs, such as summer youth employment, are an effective intervention to reduce gun violence.^45^ These programs provide youth with structured learning opportunities that help them develop critical social connections and job development skills. A study evaluating the impact of a summer youth employment program in Boston found a 35% reduction in youth violent crime involvement more than a year after the program ended.^46^ It appeared that the program was protective because it offered participants skills they needed, not simply because it offered a temporary place to stay safe. This finding supports the view that investments may help alter youth trajectories, producing long-term benefits for both individuals and communities.

## Looking Forward

Even after *Bruen*, proponents of firearm injury prevention can turn to practical intervention strategies. None of these approaches is new, but recent investments are rapidly advancing the field. Since 2020, new federal funding for CVI has flowed through COVID recovery funds, gun safety legislation, and other grants programs.^47^ Scaling up CVI will produce substantial new human infrastructure for community safety. This change coincides with recent growth in federal funding for firearm injury research, which has historically been among the most underfunded areas of public health research.^48^ Research will help CVI programs hone their approaches, and may help identify synergies between person- and place-based investments, particularly as community violence researchers adopt methods designed for understanding complex systems (*e.g.,* computer simulation modeling^49^). These countercurrents to the *Bruen* decision offer hope that community violence can be curbed, particularly in the neighborhoods suffering the worst impacts today.
